# Mutations in *CERS3* Cause Autosomal Recessive Congenital Ichthyosis in Humans

**DOI:** 10.1371/journal.pgen.1003536

**Published:** 2013-06-06

**Authors:** Franz P. W. Radner, Slaheddine Marrakchi, Peter Kirchmeier, Gwang-Jin Kim, Florence Ribierre, Bourane Kamoun, Leila Abid, Michael Leipoldt, Hamida Turki, Werner Schempp, Roland Heilig, Mark Lathrop, Judith Fischer

**Affiliations:** 1 Institute for Human Genetics, University Medical Center Freiburg, Freiburg, Germany; 2 Department of Dermatology and the Laboratory of Immunology, Hedi Chaker Hospital, Sfax University, Sfax, Tunisia; 3 Faculty of Biology, University of Freiburg, Freiburg, Germany; 4 CEA, Institut de Génomique, Centre National de Génotypage, Evry, France; 5 Department of Ophthalmology, Hedi Habib Bourguiba Hospital, Sfax University, Sfax, Tunisia; 6 Department of Cardiology, Hedi Chaker Hospital, Sfax University, Sfax, Tunisia; 7 CEA, Institut de Génomique, Centre National de Séquencage, Genoscope, Evry, France; 8 CEPH, Paris, France; 9 McGill University and Génome Québec Innovation Centre, Montréal, Canada; University of Oxford, United Kingdom

## Abstract

Autosomal recessive congenital ichthyosis (ARCI) is a rare genetic disorder of the skin characterized by abnormal desquamation over the whole body. In this study we report four patients from three consanguineous Tunisian families with skin, eye, heart, and skeletal anomalies, who harbor a homozygous contiguous gene deletion syndrome on chromosome 15q26.3. Genome-wide SNP-genotyping revealed a homozygous region in all affected individuals, including the same microdeletion that partially affects two coding genes (*ADAMTS17*, *CERS3*) and abolishes a sequence for a long non-coding RNA (*FLJ42289*). Whereas mutations in *ADAMTS17* have recently been identified in autosomal recessive Weill-Marchesani-like syndrome in humans and dogs presenting with ophthalmologic, cardiac, and skeletal abnormalities, no disease associations have been described for *CERS3* (ceramide synthase 3) and *FLJ42289* so far. However, analysis of additional patients with non-syndromic ARCI revealed a splice site mutation in *CERS3* indicating that a defect in ceramide synthesis is causative for the present skin phenotype of our patients. Functional analysis of patient skin and *in vitro* differentiated keratinocytes demonstrated that mutations in *CERS3* lead to a disturbed sphingolipid profile with reduced levels of epidermis-specific very long-chain ceramides that interferes with epidermal differentiation. Taken together, these data present a novel pathway involved in ARCI development and, moreover, provide the first evidence that CERS3 plays an essential role in human sphingolipid metabolism for the maintenance of epidermal lipid homeostasis.

## Author Summary

Autosomal recessive congenital ichthyosis (ARCI) is a heterogeneous group of human keratinization disorders mainly characterized by generalized abnormal scaling of the skin. To date, positional cloning and homozygosity mapping of families with ARCI have identified disease-associated mutations in seven genes: *ABCA12*, *ALOX12B*, *ALOXE3*, *CYP4F22*, *ICHTHYIN*, *PNPLA1*, and *TGM1*. The reported molecular mechanisms underlying disease development are related to defects in epidermal lipid pathways that interfere with terminal keratinocyte differentiation and skin barrier function. In this study we used genome-wide SNP mapping, which identified homozygous mutations in the *CERS3* (ceramide synthase 3) gene that cause a new type of ARCI. Functional analysis of a skin sample and *in vitro* differentiated keratinocytes from one patient demonstrated that mutated CERS3 impairs the synthesis of ceramides with very long-chain acyl moieties. The defect in sphingolipid metabolism disturbs the epidermal lipid profile, which leads to an abnormal terminal differentiation process. In summary, mutations in *CERS3* are causative for ARCI and illustrate the important role of ceramide synthesis in human skin physiology.

## Introduction

Autosomal recessive congenital ichthyosis (ARCI) is characterized by abnormal desquamation over the whole body due to a dysfunctional skin permeability barrier and an altered lipid composition of the skin. To date, in our collection of about 550 families presenting with ARCI we identified mutations in seven different genes, which can cause non-syndromic forms of ARCI: *ABCA12*, *ALOXE3*, *ALOX12B*, *CYP4F22*, *ICHTHYIN*, *PNPLA1*, and *TGM1* [1]–[6] in about 80% of the patients; the remaining 20% of patients are expected to carry mutations in other genes, which remain to be identified [7].

Here we present a new contiguous gene deletion syndrome affecting the sequences of *ADAMTS17*, *FLJ42289*, and *CERS3* in four patients from three consanguineous families from our collection. The clinical phenotype of the affected individuals partly corresponds to the characteristic manifestation of Weill-Marchesani (WM)-like syndrome (MIM 613195) that has been found to be caused by loss-of-function mutations in the *ADAMTS17* (a disintegrin-like and metalloproteinase with thrombospondin type-1 motif 17) gene [8], [9]. However, the present ichthyosis skin phenotype of our patients has never been reported in WM-like syndrome patients, suggesting that mutations in *FLJ42289* and/or *CERS3* (ceramide synthase 3) could be associated with this unusual form of ARCI. We therefore performed sequence analyses of the affected individuals and functional studies on mutant skin samples and keratinocytes, which were differentiated *in vitro*. In this way, we were able to characterize a new type of ichthyosis and to provide evidence for the involvement of *CERS3* mutations in the development of ARCI in humans.

## Results

### Sequence analysis of affected individuals with ARCI revealed mutations in *CERS3*

**Figure 1:**
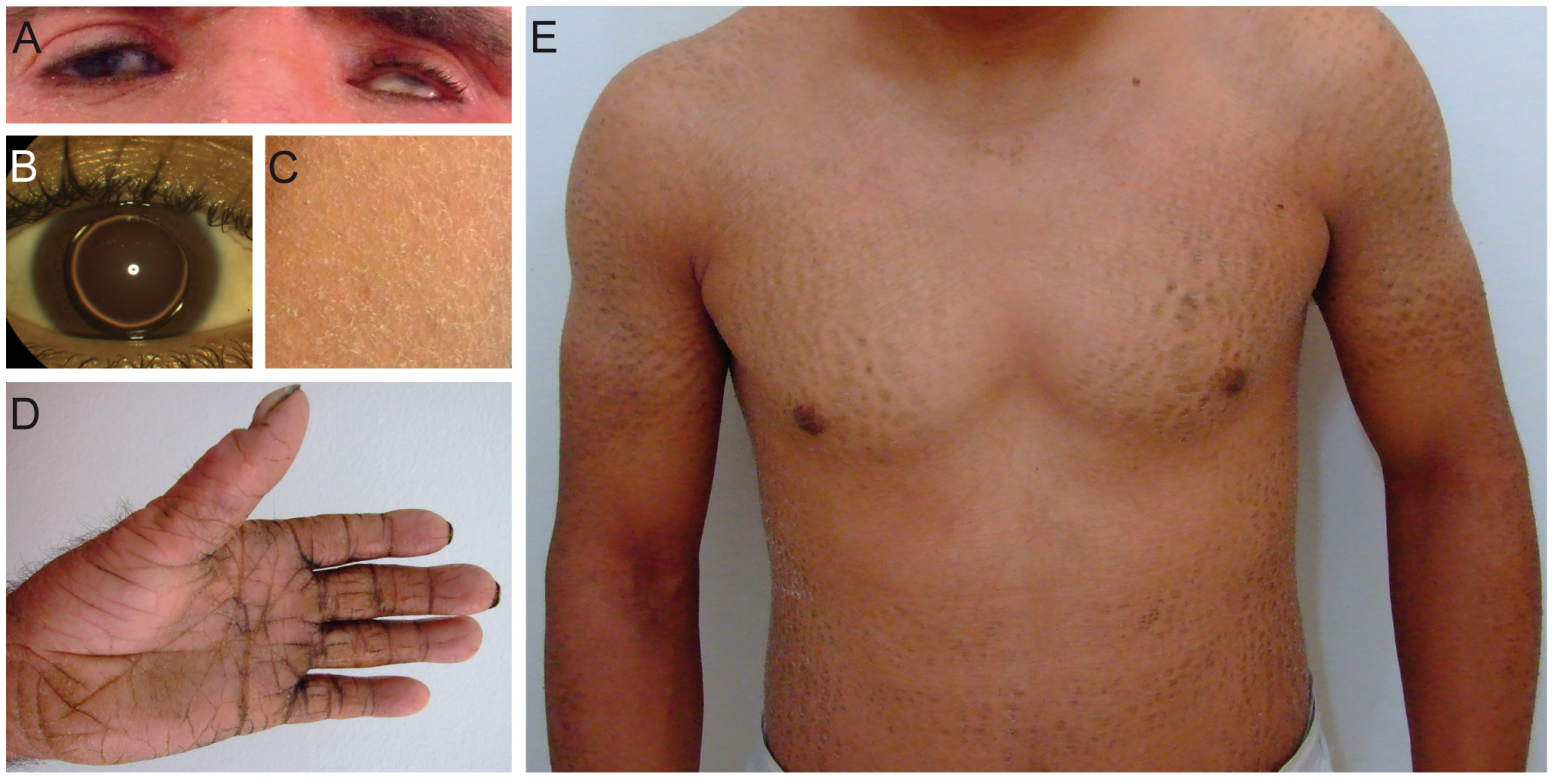
Clinical features of patients. (**A**) Patient D1 with erythema of the face and eye symptoms; (**B**) Patient S with isolated bilateral microspherophakia without ectopia or hypertonia; (**C**) Skin of the upper leg of patient S showing ichthyosiform erythroderma with large, white scales; (**D**) Palmoplantar hyperlinearity, hyperkeratosis, and brachydactyly in patient D1; (**E**) Upper part of the body in patient D2; large brownish scales on the limbs and trunk.

**Figure 2:**
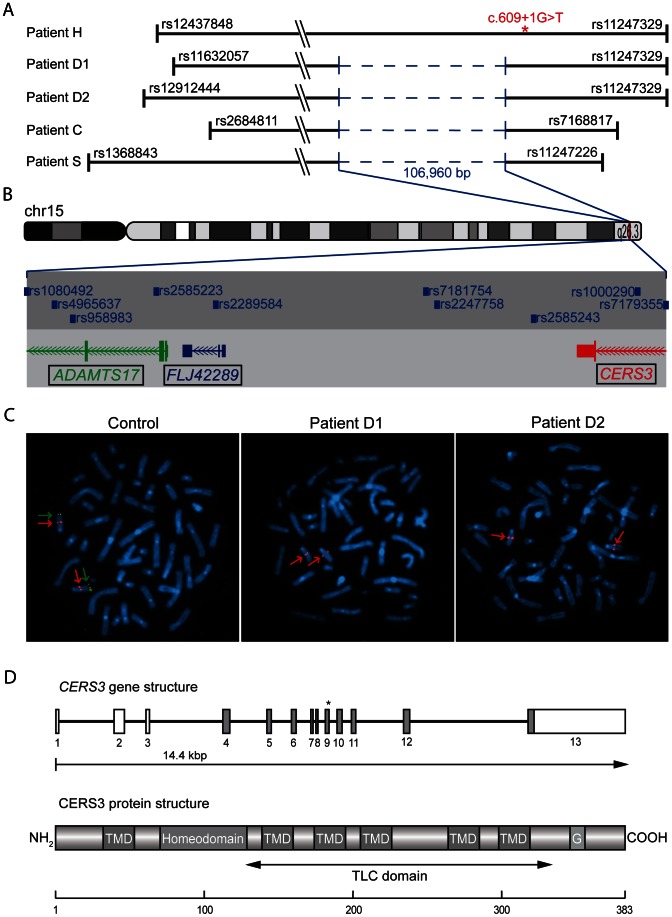
Deletion and *CERS3* mutation scheme. (**A**) Homozygous regions on chromosome 15q26.3 in patients. The smallest common interval is defined by rs2684811 in patient C and rs11247226 in patient S. The genomic deletion characterized by breakpoint spanning PCR (in blue) encompasses 106,960 bp with the borders 100,856,031 to 100,962,985 on chromosome 15 (UCSC hg19, February 2009) in patient D1, D2, C, and S. The splice site mutation in patient H is marked by an asterisk. The diagram shows the deletion and the limiting SNPs of the homozygous regions in patient H, D1, D2, C, and S (not to scale). (**B**) The scale illustration of the deleted region shows the missing SNPs and genes. The internal limit of the deletion is between SNP rs1080429 and rs7179355. (**C**) FISH signal pattern in a healthy individual shows the control signal on 15q21.2 in red (digoxigenin-labeled BAC RP11-562A8) and the signal corresponding to 15q26.3 in green (arrow). In patients D1 and D2 the green FISH signal is missing confirming the microdeletion in 15q26.3. (**D**) Diagram depicting the structure of the human *CERS3* gene and the site of mutation in exon 9 (c.609+1G>T) of patient H indicated by an asterisk. Below is illustrated the predicted structure of human CERS3 protein including transmembrane domains (TMD), homeobox, polyglutamic acid region (G), and the TLC (TRAM, LAG1 and CLN8 homology) domain.

We performed genome-wide SNP-array based homozygosity mapping in 34 consanguineous families with ARCI including three Tunisian families with a syndromic form of ARCI. The clinical features in the four patients (D1, D2, C, and S) include collodion membrane at birth evolving to generalized ARCI, but also short stature, brachydactyly with joint stiffness, microspherophakia, ectopia lentis, mitral valve defects, and multiple nevi (Table 1, Figure 1, and Text S1). These patients all shared a homozygous region on chromosome 15q26.3 with an identical haplotype in the smallest common interval of 1.67 Mb (Figure 2A). Within this region, a homozygous deletion of about 100 Kb was observed between the SNP markers rs1080492 and rs7179355 that encompasses the first three exons of *ADAMTS17*, the complete sequence of the non-coding RNA *FLJ42289*, and exon 13 of *CERS3* including the 3′UTR (Figure 2B). We confirmed the homozygous deletion in these patients using FISH (Figure 2C) and array CGH (comparative genomic hybridization) analysis (data not shown). A breakpoint spanning PCR followed by sequencing defined the genomic deletion encompassing 106,960 bp (Figure S1). Moreover, sequencing of all exons and exon/intron boundaries of the *CERS3* gene showed the absence of PCR amplification of exon 13 and therefore confirmed the genomic deletion in the patients (D1, D2, C, and S). *ADAMTS17* was not sequenced. One additional Tunisian individual (H) with isolated ARCI also showed a homozygous region on 15q26.3, but did not carry the genomic deletion described above (Figure 2A). However, sequencing of the *CERS3* gene in this patient revealed a homozygous transversion of guanine to thymine affecting the exon 9 splice donor site (c.609+1G>T) (Figure 2D). To analyze the functional effect of this mutation event we performed reverse transcription of mRNA from patient H isolated from *in vitro* differentiated keratinocytes followed by PCR amplification of the corresponding *CERS3* region using specific primers. Separation by agarose gel electrophoresis as well as sequencing of the PCR fragment demonstrated a reduced length of the PCR product due to skipping of exon 9 resulting in an in-frame deletion of 93 bp in the *CERS3* coding transcript (Figure S2). MutationTaster software calculated a result score of 0.999 for a probable disease causing mutation event [10]. In addition, this sequence variation was not found in 96 unaffected population-matched control individuals.

**Table 1:** Clinical characteristics of the patients.

Patient	D1	D2	S	C	H
**Mutation**					
Microdeletion 15q26.3	+	+	+	+	−
Splice Site Mutation *CERS3*	−	−	−	−	+
**Age in Years/Sex**	37/m	22/m	14/m	11/m	30/f
**Skin Symptoms**					
Collodion baby at birth	+	+	+	+	+
**Face**					
Erythema and fine scales	+	+	+	+	+
**Trunk**					
Erythema	+	+	+	+	+
Scales	fine white	large brown	fine white	fine	fine
**Lower Limbs**					
Erythema	+	+	+	+	+
Scales	large brown	large brown	large brown	large	large brown
Hyperlinearity of palms	+	+	+	+	+ (and soles)
Hyperkeratosis (moderate)	+	+	+	+	+
Premature aging aspect of back of hands	+	+	+	+	+
Multiple nevi	−	−	face, trunk, limbs	+	+
**Additional Symptoms**					
**Short Stature in m**	1.65	1.63	1.50	1.33	1.49
Brachydactyly	+	+	+	−	−
Joint stiffness	+	−	+	−	−
**Eye Symptoms**					
Acuity (R//L)	−	3∶10/4∶10	−	−	−
Microspherophakia	bilateral	bilateral	bilateral	bilateral	−
Myopia (R/L)	−	−9/−3,75	−6/−6	−8/−7,25	−
Cataract	+	−	−	−	−
Retinal detachment	+ (left eye)	−	Amblyopia	−	−
Glaucoma	+	−	−	−	−
**Heart**					
Tachycardia	+	−	−	−	−
Mitral valve dysplasia	+	+	−	+	−
Cardiomyopathy (dilated)	+	−	−	−	−

### *CERS3* mutations cause abnormal skin morphology

**Figure 3:**
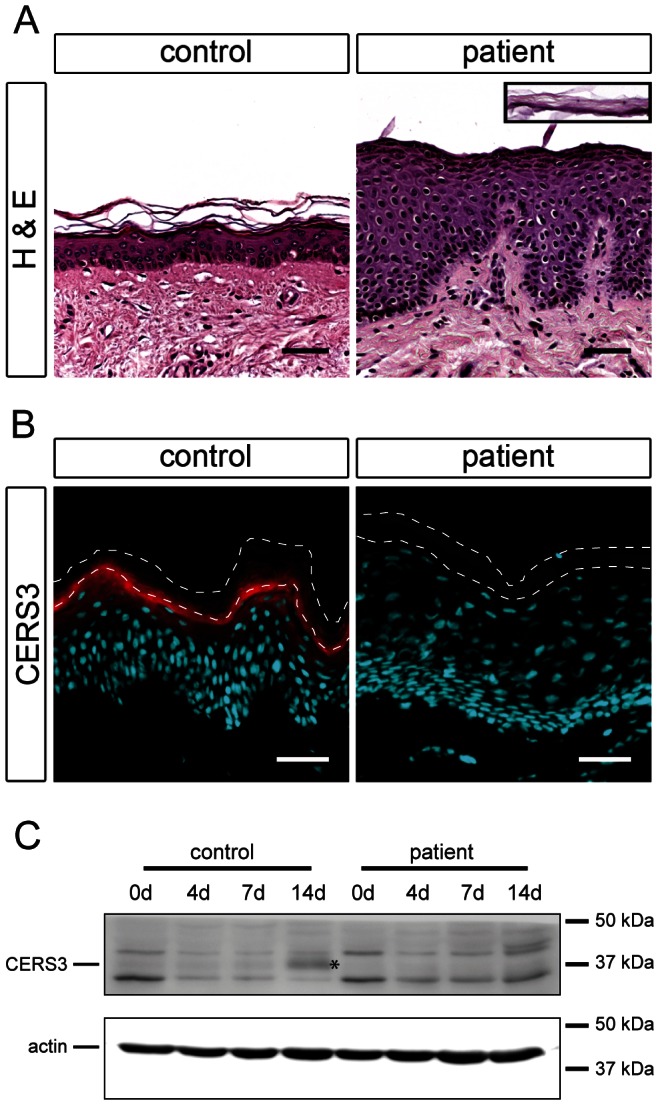
Histological analysis and CERS3 protein expression in skin biopsies and cultured keratinocytes from healthy controls and patient H. (**A**) Hematoxylin and eosin staining (H&E) shows acanthosis with a thickening of the granular layer and psoriasiform epidermal hyperplasia in the patient compared to a healthy control. The inset (same scale) shows the detached stratum corneum of the patient. Scale bars, 50 µm (**B**) Immunofluorescence staining using a specific antibody for CERS3 (red) and DAPI (blue) as nuclear counterstaining. CERS3 staining is present at the interface between the stratum granulosum and the stratum corneum in control skin but not detectable in the patient skin. The thin dashed lines indicate the interface between the stratum granulosum and the stratum corneum as well as the upper edge of the stratum corneum. Scale bars, 50 µm (**C**) Western blot analysis of CERS3 in control and patient keratinocytes before differentiation (0 d) and at day 4, 7, and 14 after induction of differentiation. CERS3 was detected at ∼37 kDa (indicated by an asterisk) using an antibody targeting the C-terminus of the protein. An antibody that recognizes actin was used as a loading control.

To investigate the role of *CERS3* mutations in the development of ichthyosis we performed histological and biochemical studies using a skin sample of patient H carrying the splice site mutation in the *CERS3* gene. Since we could not exclude that mutations in *ADAMTS17* and/or *FLJ42289* interfere with skin physiology, we did not include samples from the patients with the gene deletion syndrome in the following experiments. Histological analysis of the skin biopsy from patient H showed acanthosis with thickening of the stratum granulosum, psoriasiform epidermal hyperplasia (Figure 3A), and normal size of the detached stratum corneum (inset Figure 3A). To examine CERS3 distribution in skin we performed immunofluorescence staining of paraffin as well as frozen sections from human control biopsies. Using an antibody targeting an epitope near the N-terminus of CERS3 (amino acid 59–120) we demonstrated that the protein localizes at the interface between the stratum granulosum and the stratum corneum in the epidermis (Figure 3B and Figure S3) in accordance with data of the *Human Protein Atlas* (http://www.proteinatlas.org). In contrast, the patient's skin biopsy did not show CERS3 staining suggesting that functional protein is not present in mutant epidermis (Figure 3B). We verified these results by immunoblotting of lysates from *in vitro* differentiated control and patient keratinocytes using an antibody targeting amino acids 370–383 at the C-terminus of CERS3. Immunoblots revealed expression of the protein in control cells at the basal stage (day 0) and increased levels during progression of differentiation (Figure 3C). Concordant with the results of immunofluorescence staining, we did not detect full-length CERS3 or a truncated version of the protein in mutant keratinocytes under basal conditions as well as at later stages of keratinocyte differentiation.

### Mutated CERS3 disturbs the epidermal sphingolipid profile

**Figure 4:**
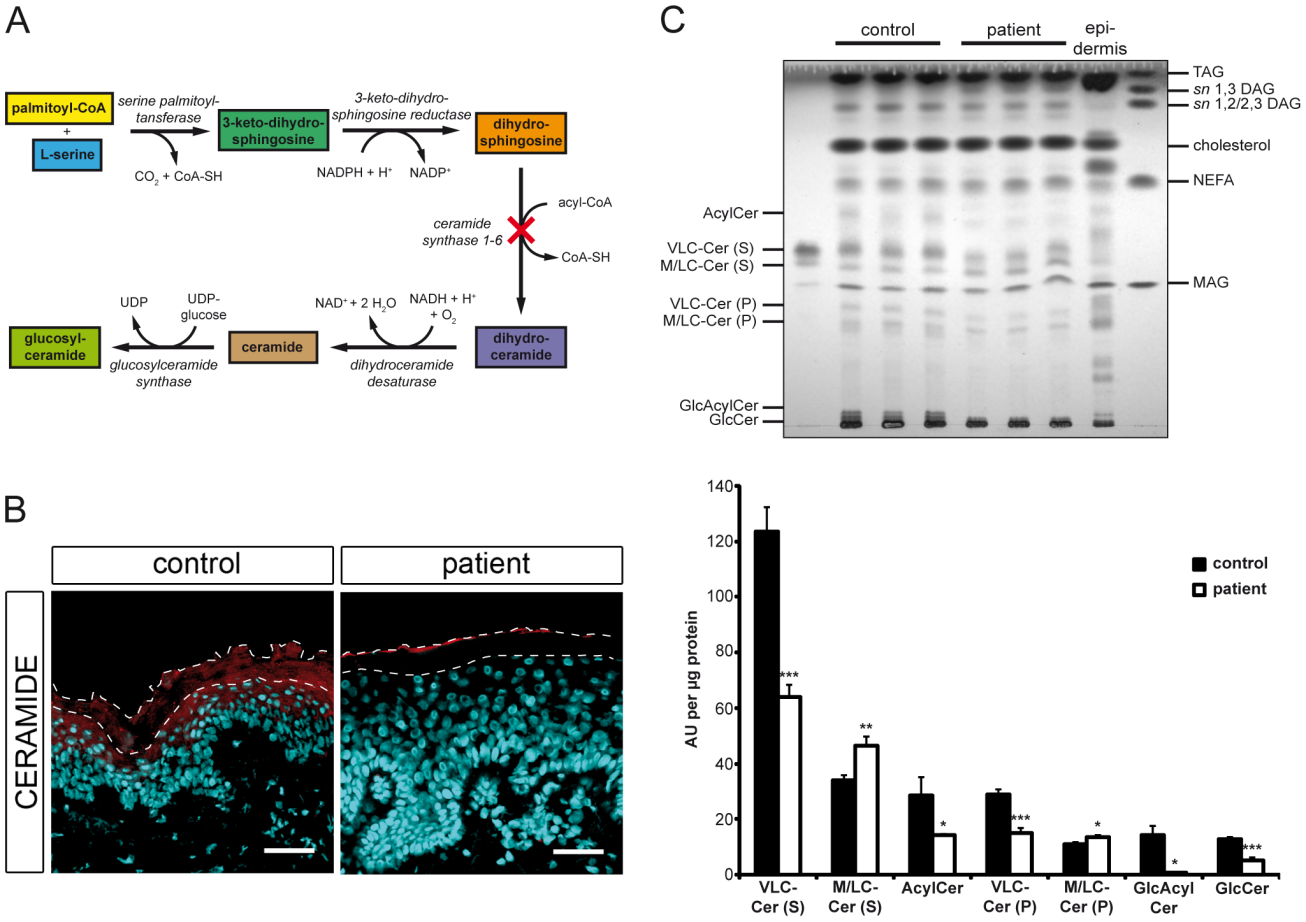
*In situ* ceramide localization and sphingolipid profile of cultured keratinocytes. (**A**) Diagram depicting the *de novo* synthesis pathway of (glucosyl)ceramides. Loss-of-function of ceramide synthase 1–6 results in an impaired *N*-acylation of dihydrosphingosine as indicated by a cross. (**B**) Confocal microscopy images of healthy control and patient H skin biopsies immunostained with an antibody targeting ceramides (red) with DAPI (blue) as nuclear counterstaining. Ceramides localize to the stratum granulosum and the stratum corneum in healthy control skin. Note the loss of ceramide staining in patient's skin. We observed immunostaining of ceramides in the uppermost layer of the mutant stratum corneum, which results from unspecific binding of the secondary antibody. The thin dashed lines indicate the interface between the stratum granulosum and the stratum corneum as well as the upper edge of the stratum corneum. Scale bars, 50 µm (**C**) Upper panel: TLC of lipid extracts from healthy control and patient H keratinocytes 14 days after induction of differentiation. Lipids corresponding to 400 µg of cellular protein were extracted from cultures, separated twice by TLC using the solvent system chloroform/methanol/glacial acetic acid (190/9/1 v/v/v), and quantified after carbonization. An epidermal lipid extract of a healthy control individual was used as a reference. Lower panel: Data are presented as mean values +S.D. of triplicate samples and are representative for three independent experiments. Statistical significance was determined by unpaired two-tailed Student's *t*-test (* *p*<0.05, ** *p*<0.01, *** *p*<0.001). Ceramide species are classified according to the sphingoid base (S, sphingosine or P, phytosphingosine). Abbreviations: AcylCer, acylceramides; DAG, diacylglycerols; GlcAcylCer, glucosylacylceramides; GlcCer, glucosylceramides; MAG, monoacylglycerols; M/LC-Cer, middle and long-chain ceramides; NEFA, non-esterified fatty acids; TAG, triacylglycerols; VLC-Cer, very long-chain ceramides.

Since CERS3 generates epidermis-specific ceramides by *N*-acylating dihydrosphingosine with acyl-CoAs ranging from long to very long aliphatic chains (C18–C28) [11], [12] (Figure 4A), we examined whether mutated CERS3 affects the localization and concentration of ceramides in human epidermis using a commercially available antibody targeting ceramides [13]. Immunofluorescence analysis on frozen sections of a control skin biopsy demonstrated the presence of ceramides in the stratum granulosum and stratum corneum, consistent with the localization of CERS3. In the skin biopsy of the patient carrying the *CERS3* splice donor site mutation, however, we detected a massive reduction of ceramides in these layers (Figure 4B). To study the disturbed sphingolipid profile of the patient keratinocytes in detail, we performed TLC analysis of lipid extracts from differentiated control and mutant cells (Figure 4C and Figure S4). Compatible with the results of immunofluorescence staining, lipid extracts of patient keratinocytes compared to control samples exhibited a marked decrease of very long-chain (VLC) ceramides with sphingosine (−48.2%) and phytosphingosine (−47.9%) as sphingoid base as well as significantly decreased levels of acylceramides (−49.9%), glucosylacylceramides (−95.9%), and glucosylceramides (−60.2%). In contrast, levels of ceramides with middle to long-chain acyl moieties (C16–20) were slightly but significantly increased (1.4-fold for sphingosine and 1.2-fold for phytosphingosine as sphingoid base) in patient keratinocytes compared to control samples.

### Mutations in *CERS3* lead to an abnormal terminal differentiation

**Figure 5:**
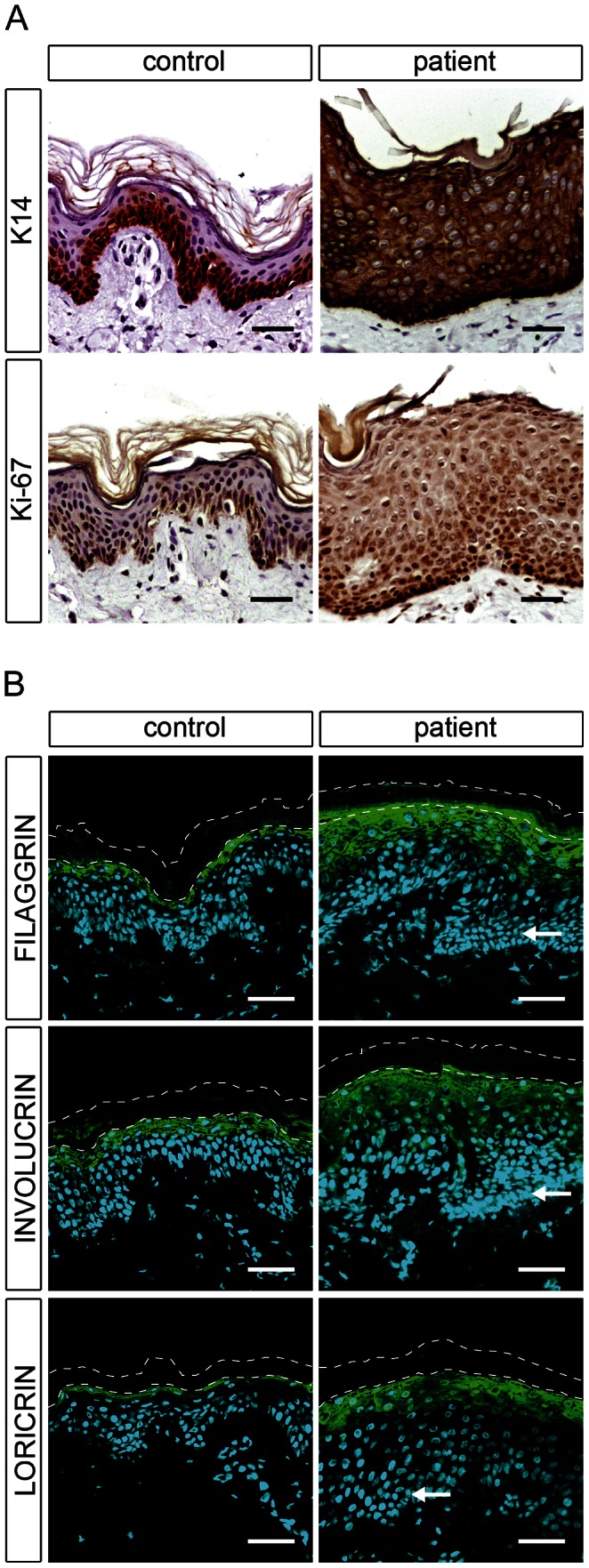
Epidermal differentiation in healthy control and patient H. (**A**) Light microscopy images of skin biopsies from control and patient H immunolabeled with antibodies specific for keratin 14 (K14) and Ki-67 with hematoxylin as nuclear counterstaining reveal an abnormal differentiation process in patient skin. (**B**) Confocal microscopy images of the same control and patient skin biopsies immunostained with antibodies specific for filaggrin, involucrin, and loricrin with DAPI (blue) as nuclear counterstaining. The patient skin biopsy shows a thickening of the stratum granulosum compared to the healthy control. Arrows indicate hyperplastic basal cells in the patient skin. The thin dashed lines indicate the interface between the stratum granulosum and the stratum corneum as well as the upper edge of the stratum corneum. Scale bars, 50 µm.

To examine the effect of *CERS3* mutations on epidermal differentiation, we performed immunofluorescence and immunohistochemical analysis using established differentiation markers for keratinocytes. In healthy control skin, immunohistochemical staining showed that K14, a marker of undifferentiated keratinocytes, was almost exclusively present in the stratum basale arranged as a one- or two-cell layer (Figure 5A, upper panel). In a skin sample of patient H, however, K14 staining expanded to upper cell layers. During differentiation, keratinocytes from the basal layer gradually migrate upwards forming the upper layers of the epidermis. Thus, an expansion of the basal layer in mutant skin could be a result of an increased proliferation and/or delayed terminal differentiation of keratinocytes. However, immunolabeling of the proliferation marker Ki-67 in control and patient skin corresponded to the K14 expression pattern, indicating a delayed epidermal differentiation and an increased number of proliferating cells in mutant skin (Figure 5A, lower panel). Immunofluorescence staining for involucrin, loricrin, and filaggrin, markers for terminally differentiated keratinocytes, revealed a thickening of the upper stratum spinosum and stratum granulosum in patient skin compared to control samples. Together, these observations suggest that mutated CERS3 affects the terminal differentiation process in human skin (Figure 5B).

## Discussion

We report four patients with a genomic microdeletion on chromosome 15q26.3 that partially affects the sequences of *CERS3* and *ADAMTS17* and abolishes the sequence of the non-coding RNA *FLJ42289*. We observed the same haplotype with an identical genomic deletion in these patients and since they originate from the same geographical region in Tunisia, we concluded that the present contiguous gene deletion syndrome is due to a founder effect. The loss of exon 13 in *CERS3* with the 3′UTR and the first three exons of *ADAMTS17* including the 5′UTR and the start codon predict a reduced mRNA transcript stability of both genes that renders protein translation unlikely. Moreover, the genomic deletion could lead to a fusion protein of CERS3 with ADAMTS17 resulting in a stop codon in exon 4 of *ADAMTS17*. If so, the premature termination codon in the predicted fusion gene suggests an enhanced transcript instability triggered by nonsense-mediated mRNA decay [14]. Exon 13 of *CERS3* corresponds to the C-terminal amino acids 334–383 that are predicted to form a coiled-coil structure (amino acids 335–355, http://www.ensembl.org). This domain is found in many other proteins involved in important biological functions such as gene regulation (e.g. transcription factors) or vesicular transport [15], [16]. Moreover, because of their specific interaction, coiled-coil structures are essential for protein-protein interactions thereby facilitating dimerization (e.g. leucine zipper) [17], [18]. Whether CERS3 interacts with DNA or with other proteins via its coiled-coil motif is not known so far. However, the C-terminal 50 amino acids of CERS3 are evolutionary highly conserved among mammals (homology 62–96%) indicating an important role of this domain for protein function.

Since the partial deletion of *ADAMTS17* and *CERS3* seems to have a pathologic effect only when both alleles are affected, and since the parents of our patients have no obvious disease symptoms, we conclude an autosomal recessive mode of inheritance. Thus, the present contiguous gene deletion syndrome leads to two independent diseases (ARCI and WM-like syndrome). Currently, it is unclear whether the deletion of the non-coding RNA *FLJ42289* contributes to the phenotype of the syndrome.

Recently, Morales *et al.* [8] described a related WM-like syndrome phenotype in eight individuals from Saudi Arabian families with mutations in *ADAMTS17*, who displayed many of the key features of WM-like syndrome, including lenticular myopia, ectopia lentis, glaucoma, spherophakia, and short stature, but none of these patients had the characteristic brachydactyly or decreased joint flexibility of WM-like syndrome. However, in our four patients with the homozygous 15q26.3 microdeletion in which the first three exons of *ADAMTS17* are missing, three of them show a brachydactyly and two of them have reduced joint flexibility (Table 1, Figure 1, and Text S1). This observation is coherent with phenotypic variability found in most genetic syndromes and underlines the importance of clinical reports in rare genetic disorders.

The identification of the *CERS3* splice donor site mutation, c.609+1G>T, in an additional patient (H) with isolated ARCI confirmed that mutations in *CERS3* are causative for the skin anomalies in this form of contiguous gene deletion syndrome. The splice site mutation leads to the loss of exon 9 in the *CERS3* coding transcript. This sequence region corresponds to a transmembrane domain (amino acid 174–194) of CERS3 that is thought to be essential for proper membrane topology of the protein. Thus, the loss of this structure would most likely affect protein localization as well as stability. Immunohistochemistry using an antibody that recognizes an N-terminal epitope of CERS3 demonstrated the presence of the protein at the interface between the stratum granulosum and the stratum corneum in control skin but was not detectable in the patient H. CERS3 belongs to a family of enzymes encoded by the *CERS* genes (*CERS1*–6 in mammals) that were originally referred to as *LASS 1–6* (longevity assurance homolog 1–6) based on their homology to the yeast protein, longevity assurance 1 (LAG1p) [19]–[21]. All mammalian CERS are integral membrane proteins of the endoplasmic reticulum that catalyze the acylation of the free amine nitrogen of the sphingoid long chain base to form an amide bond (*N*-acylation, Figure 4A) [22], [23]. In this reaction each CERS prefers acyl-CoAs of specific carbon chain length to synthesize ceramides [11], [12], [19]–[21], [24]–[26]. Of the six known mammalian CERS, CERS3 shows the broadest substrate spectrum utilizing acyl-CoAs of 18 to 28 carbon chain lengths [11], [12]. Quantitative RT-PCR revealed that *CERS3* mRNA is mainly expressed in testis and epidermis, and its expression in these tissues strongly increases upon differentiation [27], [28]. Thus, CERS3 contributes to the fatty acid composition and concentration of ceramides in these tissues. In skin sections of the patient carrying the splice site donor mutation we did not detect ceramides in the epidermis by immunohistochemistry using a commercially available antibody (Figure 4B). Thus, we conclude that the patients' ichthyosis skin phenotype results from mutations in *CERS3* that significantly impair the epidermal ceramide synthesis, in particular the synthesis of (glucosyl)acylceramides. Using TLC analysis, however, we identified VLC ceramides in *in vitro* differentiated patient keratinocytes (Figure 4C) suggesting that the mutated CERS3 may harbor residual enzymatic activity. In addition, some limited synthesis of VLC ceramides may occur in patient keratinocytes by the enzymatic activity of other members of the CERS family that compensate for the CERS3 defect. Indeed, CERS2 as well as CERS4 show epidermal expression and substrate specificities toward acyl-CoAs with acyl chain lengths of 22 to 26 carbon atoms [12], [29].

In mammalian epidermis, ceramides represent the most abundant components of the stratum corneum lipid, which forms a barrier against the penetration of chemicals and pathogenic microorganisms as well as the unregulated loss of water [30]. There are at least eleven different ceramide species in human skin, differing in their fatty acid composition as well as their sphingoid base [29]. To date, the molecular details of sphingolipid metabolism resulting in this huge structural diversity of epidermal ceramides as well as their role in terminal differentiation of keratinocytes have not been extensively studied. However, profound knowledge of these pathways and their regulation is of great interest for the understanding of skin physiology and to provide novel targets and strategies for the treatment of ichthyosis and possibly other lipid-associated disorders of the skin. In this context, the metabolism of (glucosyl)acylceramides may play a central role in epidermal lipid pathways [31].

Acylceramides are epidermis-specific sphingolipids carrying amide-linked VLC fatty acids with a terminal hydroxyl group that is further esterified with linoleic acid. In mammalian skin, these ceramides are an absolute prerequisite for the formation of an intact stratum corneum and the water permeability barrier [31]. Accordingly, loss-of-function of enzymes involved in acylceramide metabolism result in cutaneous barrier abnormalities such as those found in human diseases and corresponding mouse models like ELOVL4-deficiency (MIM 614457) [32]–[34] or Chanarin-Dorfman syndrome (MIM 275630) [35], [36]. The skin of these patients and mutant mice does not exhibit detectable levels of acylceramides and displays a delayed terminal differentiation process that is similar to the one observed in CERS3-deficient skin, arguing for a fundamental role of acylceramides in keratinization. The importance of ceramides and their metabolism for cellular proliferation and differentiation is also evident in other organs and cell types. Very recently, Jennemann *et al.* [37] demonstrated that glucosylceramides are essential for intestinal epithelial differentiation to maintain the reabsorption function of enterocytes.

Our data resemble in many ways the skin phenotype of mice with targeted disruption of the *Cers3* gene [12]. In contrast to affected patients of our families, however, *Cers3*-null mice die shortly after birth due to drastically reduced levels of VLC ceramides leading to a dysfunctional water permeability barrier and rapid dehydration of the animals [12]. This interesting phenotypic difference suggests that mice are more sensitive to barrier defects than humans probably due to a disadvantageous ratio of body volume to skin surface provoking increased dehydration. Notably, similar skin barrier defects associated with early postnatal death are also present in other mouse models for human skin disorders like Chanarin-Dorfman syndrome [36] or NISCH syndrome (MIM 607626) [38]. Future detailed studies addressing skin integrity and barrier recovery of patients with *CERS3* mutations will be required to shed light on the mechanisms involved in disease development in humans.

In summary, our study reports a contiguous gene deletion syndrome that identifies *CERS3* as another ARCI-associated gene in humans. We present functional evidence demonstrating that CERS3 is crucial for the synthesis of VLC ceramides in human skin to maintain epidermal lipid homeostasis and terminal differentiation. Therefore, we suggest that the application of lotions supplemented with VLC ceramides, especially acylceramides onto the skin of affected patients would be a promising therapeutic approach to treat skin symptoms in patients with keratinization disorders.

## Materials and Methods

### Patients

We obtained blood samples from 34 consanguineous ARCI families in collaboration with clinicians and the support of Généthon (Evry, France). DNA was extracted from whole blood according to standard procedures after written informed consent from all patients and family members who participated in the study. The medical ethics committee of AFM/Généthon approved the study.

### Genotyping and sequencing

Genome-wide SNP-genotyping was carried out in a total of 34 consanguineous families (120 individuals) using a human SNP array (Illumina 370k Quad, San Diego, CA). After quality control, the genotyping data were filtered for homozygous regions larger than 1 Mb. Both DNA strands from all subjects and controls were sequenced for the entire coding region and the exon/intron boundaries using standard protocols. Primers for *CERS3* flanking the coding exons were designed with primer3.

### FISH analysis

#### Chromosome preparation

Standard chromosome preparation methods were applied to peripheral blood lymphocyte cultures of two affected individuals (patient D1 and D2) according to standard methods with minor modifications [39]. Slides carrying interphase cells and metaphase spreads were dehydrated in ice-cold ethanol (70%, 90% and 100% each for 3 min) then air dried and stored at −80°C. Before *in situ* hybridization, cells were dehydrated again (70%, 90% and 100% each for 3 min) and then air dried.

#### FISH probe generation

Long-range PCR was applied to BAC-clone RP11-243L8. The sequence between the SNP markers rs1080492 and rs1000290 was extracted from GRCh37/hg19 assembly (positions chr15: 100,862,474–100,949,609) and masked for repeats using RepeatMasker (http://www.repeatmasker.org). PCR primers were designed in unmasked regions to generate amplicons depleted in repeats, simple sequences, and stretches of very high GC content. Long and accurate PCR reactions were performed on 2 ng of DNA of BAC RP11-243L8, which encompasses the target region, using LA-Taq DNA Polymerase (TaKaRa, Otsu, Shiga, Japan). The cycling program was: 1) 5 min at 96°C; 2) 15 cycles of 2 sec at 94°C, 20 sec at specific annealing temperature (AT) and a specific elongation time (ET) at 68°C; 3) 15 more cycles with 15 sec extension per cycle in ET and 4) 10 min of final elongation at 72°C. Specific PCR products were purified by ChromaSpin 400 columns (Clontech, Mountain View, CA). Amplicons specificities are: A: AT = 62°C; ET = 15 min. A.fwd = 5′-TGG ATT TGA GAT GTC CTA AAT CTA CTC CAG AC-3′; A.rev = 5′-CTG TTC CCA GAC TTT TCT AGT TAT ACA TCA GC-3′; Ba: AT = 60°C; ET = 8 min. Ba.fwd = 5′-CTC TAG GAT CAA GTC CAA ATG CCT TCG-3′; Ba.rev = 5′-TCA AAG TGT ATG TCA CGG GAG TAC CTG-3′; Bb: AT = 60°C; ET = 8 min. Bb.fwd = 5′-GAG AAC GCA GAG GGA AGG TAG C-3′; Bb.rev = CCA TGT TAA CAG GTG CTG GAA GTT GCT G-3′; C AT = 62°C; ET = 10 min C.fwd = 5′-CCT GAG ATG CAA TTC ATT GGT TCC TGG-3′; C.rev = 5′-TCT GTG TAT TTC CGA TAC TAG CAA CCA AGG-3′. All FISH-assays were performed on metaphase spreads following the protocol of Schempp *et al.* [40] Prior to FISH, the air dried slides were treated with RNase followed by pepsin digestion as described by Ried *et al.* [41] Chromosome *in situ* suppression (CISS) was applied to biotin-labeled long-range PCR products from BAC RP11-243L8 and digoxigenin-labeled RP11-562A8 as a control located on 15q21.2. For two-color detection, double-hybridization experiments were performed with biotinylated and digoxigenin-labeled probes. Biotinylated probes were detected with FITC-conjugated avidin and digoxigenin-labeled probes with anti-digoxigenin-mouse antibodies (Sigma-Aldrich, St. Louis, MO) followed by TRITC-conjugated goat anti-mouse antibodies (Sigma-Aldrich). After FISH, slides were counterstained with DAPI (4′,6-diamidino-2-phenylindole; 0.14 µg/ml) and mounted in Vectashield (Vector Laboratories, Burlingame, CA).

#### Fluorescence microscopy and imaging

Preparations were evaluated using a Zeiss Axiophot epifluorescence microscope equipped with single-bandpass filters for excitation of red, green, and blue (Chroma Technologies, Rockingham, VT). During exposures, only excitation filters were changed allowing for pixel-shift-free image recording. Metaphases were photographed by a cooled CCD camera coupled to the microscope. The DAPI, TRITC, and FITC images were merged using image software Adobe Photoshop.

### Array CGH

Genomic DNA from 4 individuals of family C (one patient, one non-affected brother, and parents) was analyzed on Microarray Sure Print G3 Human CGH+SNP chip 4x180K (Agilent, Santa Clara, CA). The probes were annotated against NCBI Build 37 (UCSC hg19, February 2009). The fragmentation, labelling, and purification of test DNA (reference DNA: HapMap sample of known genotype) as well as hybridization and washing was performed according to Agilent's protocol. The scanning step was performed with Agilent high resolution C scanner (3μ) and Agilent Feature Extraction software. Agilent Cytogenomics software 2.0.6.0 was used for imaging.

### Deletion-specific PCR spanning the genomic breakpoint

Deletion-specific PCR was performed using Phusion High-Fidelity DNA Polymerase (Finnzymes Espoo, Finland) according to the manufacturer's instructions (annealing temperature 60°C). The following primer pair D.fwd = 5′-AAT GCC TCT GAG GAG CAA GG-3′ and D.rev = 5′-GGA ATG TGA ATT AGT TTG GCC A-3′ was used to obtain a breakpoint spanning 1.2 kb PCR-product. The PCR-product was sequenced using PCR primers.

### Histological and immunohistochemical analysis

For histology, samples were collected in PBS, fixed in 4% PFA, embedded in paraffin, sectioned to 7 µm, and stained with hematoxylin and eosin. Immunostaining was performed using the Vectastain ABC Kit and the Avidin/Biotin Blocking Kit (both from Vector Laboratories) following the manufacturer's guidelines. Antigen retrieval was performed at pH 9.0 in a pressure cooker. A rabbit monoclonal antibody to cytokeratin 14 (1∶300 dilution, AC-0058, Epitomics, Burlingame, CA) and rabbit monoclonal antibody to Ki-67 (1∶300, AC-0009, Epitomics) were used as primary antibodies and a biotinylated goat anti-rabbit IgG antibody (1∶200, BA-1000, Vector Laboratories) as secondary antibody. Stained samples were examined using a Zeiss Axioskop 40 microscope with a Zeiss CCD camera.

### Immunofluorescent analysis and confocal microscopy

Skin biopsies were fixed in 4% PFA, embedded in Tissue-Tek O.C.T. Compound (Sakura Finetek, Torrance, CA), shock frozen in liquid nitrogen, and sectioned to 8 µm. For double immunofluorescence staining, the skin biopsy was fixed in 4% PFA and embedded in Paraplast Plus (Leica Microsystems, Wetzlar, Germany) followed by sectioning to 8 µm. Antigen retrieval was performed at pH 6.0 in a pressure cooker. The following commercial antibodies were used: rabbit polyclonal antibody to an N-terminal epitope of CERS3 (1∶100, HPA006092, Sigma-Aldrich), mouse monoclonal antibody to ceramide (1∶100, MAB_0011, Glycobiotech, Kükels, Germany), rabbit polyclonal antibody to loricrin (1∶500, ab24722, Abcam, Cambridge, UK), mouse monoclonal antibody to involucrin (1∶200, I9018, Sigma-Aldrich), and mouse monoclonal antibody to filaggrin (1∶200, SPM181, Abcam) as primary antibodies and Alexafluor 488 donkey antibody to mouse IgG (1∶200, 715-545-150, Jackson ImmunoResearch, West Grove, PA), Alexafluor 594 donkey antibody to rabbit IgG (1∶200, 711-585-152, Jackson ImmunoResearch), DyLight 488 donkey antibody to rabbit IgG (1∶150, 711-485-152, Jackson ImmunoResearch), and Alexafluor 546 goat antibody to mouse IgM (1∶200, A21045, Invitrogen, Carlsbad, CA) as secondary antibodies. Confocal images were captured and analyzed with an Olympus Fluoview FV1000 laser scanning confocal microscope or with a Carl Zeiss Axioplan 2 and a Photometrics CCD camera for non-confocal images.

### Keratinocyte cell culture

Primary cultures of keratinocytes were prepared according to standard protocols from skin biopsies of a control individual and patient H carrying the splice site mutation. Cells were cultured at 37°C in a humidified atmosphere with 5% CO_2_ in defined keratinocyte serum-free medium (EpiLife, Invitrogen) containing human keratinocyte growth supplement (HKGS, Invitrogen), 100 IU/ml penicillin, and 100 µg/ml streptomycin. Epidermal differentiation was induced in keratinocyte cultures according to Breiden *et al.* [42] with minor changes to promote epidermal lipid synthesis. In brief, confluent cultures were maintained in medium supplemented with 1.1 mM CaCl_2_, 30 µM palmitic acid, 25 µM oleic acid, 15 µM linoleic acid, and 10 µM lignoceric acid for 14 days with medium changes every second day. Fatty acids (Sigma-Aldrich) were complexed to fatty acid-free BSA (Sigma-Aldrich) using a ratio of 3∶1.

### Reverse transcription PCR and cDNA sequencing

Total RNA was extracted from keratinocytes before differentiation and at day 4, 7 and 14 after induction of differentiation with TRIzol Reagent (Invitrogen) according to manufacturer's protocol. 1 µg of RNA was transcribed into cDNA using M-MuLV Reverse Transcriptase (Biozym Scientific, Hessisch Oldendorf, Germany), poly d(T) primers (Sigma-Aldrich) and random hexamers (Invitrogen) according to manufacturer's guidelines. A PCR was performed using Taq Polymerase (Qiagen, Hilden, Germany) and primers localized in exon 8 and 10 of *CERS3* (forward: GGA ATG GCT ATC CCA AAC AG; reverse: GAC TCC AGC CAA ATG TCA GC). PCR fragments were separated by 2% TAE agarose gel electrophoresis according to standard laboratory protocols. PCR primers were used to sequence DNA fragments.

### Western blot analysis

At day 0, 4, 7 and 14 after induction of keratinocyte differentiation, cells were collected in RIPA buffer (150 mM NaCl, 10 mM Tris-HCl, pH 7.4, 0.1% SDS, 1% Triton X-100, 1% sodium deoxycholate and 5 mM EDTA) and lysed by sonication with a Branson Sonifier Cell disruptor B15 (output control 1, duty cycle 10%). After centrifugation at 1,000×g for 5 min to pellet nuclei and unbroken cells, the protein content was measured using BCA reagent (Pierce/Thermo Scientific, Waltham, MA) and BSA as standard. 50 µg of protein were separated by 10% SDS-PAGE according to standard laboratory protocols, blotted onto polyvinylidene difluoride membrane (Carl Roth, Karlsruhe, Germany) and hybridized with a goat polyclonal antibody raised against the C-terminus of CERS3 (1∶500 dilution, AP16822PU-N, Acris Antibodies, Herford, Germany) or a rabbit polyclonal antibody raised against actin (1∶1,000 dilution, A2066, Sigma-Aldrich). Specifically bound immunoglobulins were detected in a second reaction using HRP-conjugated anti-goat or anti-rabbit IgG antibodies (1∶10,000 dilution) and visualized by enhanced chemiluminescence detection (ECL, Amersham Biosciences, Buckinghamshire, UK).

### Lipid analysis

Total lipids were extracted from differentiated keratinocytes with chloroform/methanol/glacial acetic acid (66∶33∶1 v/v/v) and collected from the organic phase after addition of 1/5 volume of water and centrifugation at 2,400×g for 15 min. To prepare an epidermal lipid reference standard for TLC, epidermis from a healthy control individual was separated from dermis by incubating full thickness skin (15×15 mm) dermis-side down in 0.25% Trypsin-EDTA (PAA Laboratories, Pasching, Austria) at 4°C over night and total lipids were extracted from epidermis as described above. For TLC, total lipids were dried in a stream of nitrogen, reconstituted in chloroform, and spotted onto a thin-layer silica gel 60 plate (Merck, Whitehouse Station, NJ). Epidermal ceramide species were separated twice using chloroform/methanol/glacial acetic acid (190/9/1 v/v/v) as solvent system [43]. To separate polar lipids (glucosyl(acyl)ceramides) chromatograms were developed using the solvent system chloroform/methanol/water (70/30/5 v/v/v) [42]. Lipid spots were visualized by carbonization after spraying the chromatograms with 10% CuSO_4_ (w/v) and 10% H_3_PO_4_ (v/v) and heating them to 150°C for 20 min. Developed chromatograms were photographed and signals were quantified using Quantity One 1-D Analysis software (Bio-Rad Laboratories, Hercules, CA). Lipid spots of acylceramides and glucosyl(acyl)ceramides were identified according to Breiden *et al.* [42] using the epidermal lipid reference standard. After lipid extraction, cells were solubilized in 0.1% (w/v) SDS and 0.3 N NaOH at 65°C over night, and the protein content was determined using BCA reagent (Pierce/Thermo Scientific) and BSA as standard.

## Supporting Information

Figure S1 **Detailed breakpoint information and deleted elements in patients D1, D2, C, and S.** The upper panel shows the wild-type *ADAMTS17* sequence in green, aligned with the deletion junction sequence in green and red, and the wild-type *CERS3* sequence in red. The coordinates of both sequences on chromosome 15 are shown according to UCSC hg19, February 2009. The junction sequence is indicated by bold characters corresponding to *ADAMTS17* (left part in green) and *CERS3* (right part in red). The junctional CCT is highlighted in grey. The lower panel illustrates the deleted region containing 5′UTR and exon 1–3 of *ADAMTS17*, the complete sequence of *FLJ42289*, and exon 13 of *CERS3* with 3′UTR.
(TIF)

Figure S2 **RT-PCR analysis and sequencing of** ***CERS3*** **cDNA.** (**A**) RT-PCR analysis of *CERS3* mRNA from control and patient H keratinocytes before differentiation (0 d) and at day 4, 7, and 14 after induction of differentiation. Primers located in exon 8 and 10 of *CERS3* were used to determine the splicing pattern. The 213 bp-sized DNA fragment from control keratinocytes corresponds to the full-length *CERS3* coding transcript. The 120 bp-sized DNA fragment of patient H keratinocytes represent a novel *CERS3* coding transcript lacking exon 9 due to the splice donor site mutation of exon 9 (c.609+1G>T). (**B**) The sequencing of *CERS3* cDNA from healthy control and patient keratinocytes, which were differentiated *in vitro* for 14 days revealed an in-frame deletion of exon 9 in patient H (indicated by an arrow).
(TIF)

Figure S3 **Localization of CERS3 protein in human healthy skin.** Confocal microscopy images of double immunostained human skin paraffin section of a healthy individual for (**A**) the stratum granulosum marker FLG (filaggrin) (green) with DAPI as nuclear counterstaining (blue) and for (**B**) CERS3 (red), which localizes to a narrowly restricted apical layer of the epidermis. (**C**) The merged picture shows the co-localization of CERS3 and filaggrin at the interface between the stratum granulosum and the stratum corneum in the epidermis. The thin dashed lines indicate the interface between the stratum granulosum and the stratum corneum as well as the upper edge of the stratum corneum. Scale bars, 25 µm.
(TIF)

Figure S4 **TLC analysis of lipid extracts from healthy control and patient H keratinocytes 14 days after induction of differentiation.** (**A**) Lipids corresponding to 400 µg of cellular protein were extracted from cultures, separated by TLC using chloroform/methanol/water (70/30/5 v/v/v), and quantified after carbonization. Polar lipids (GlcCer and GlcAcylCer) were identified according to Breiden *et al.* [42] using an epidermal lipid extract of a healthy control individual as reference. (**B**) Data are presented as mean values +S.D. of triplicate samples and are representative for three independent experiments. Statistical significance was determined by unpaired two-tailed Student's *t*-test (* *p*<0.05, *** *p*<0.001). Abbreviations: CholSO_4_, cholesterol sulfate; GlcAcylCer, glucosylacylceramides; GlcCer, glucosylceramides; MAG, monoacylglycerols; NEFA, non-esterified fatty acids.
(TIF)

Text S1 **Detailed clinical descriptions of Tunisian patients D1, D2, S, C, and H are included in Text S1.**
(DOC)

## References

[1] Lefévre C, Audebert S, Jobard F, Bouadjar B, Lakhdar H, et al (2003) Mutations in the transporter ABCA12 are associated with lamellar ichthyosis type 2. Hum Mol Genet 12: 2369–2378. 12915478 10.1093/hmg/ddg235

[2] Jobard F, Lefèvre C, Karaduman A, Blanchet-Bardon C, Emre S, et al (2002) Lipoxygenase-3 (ALOXE3) and 12(R)-lipoxygenase (ALOX12B) are mutated in non-bullous congenital ichthyosiform erythroderma (NCIE) linked to chromosome 17p13.1. Hum Mol Genet 11: 107–113. 11773004 10.1093/hmg/11.1.107

[3] Lefèvre C, Bouadjar B, Ferrand V, Tadini G, Mégarbané A, et al (2006) Mutations in a new cytochrome P450 gene in lamellar ichthyosis type 3. Hum Mol Genet 15: 767–776. 16436457 10.1093/hmg/ddi491

[4] Lefèvre C, Bouadjar B, Karaduman A, Jobard F, Saker S, et al (2004) Mutations in ichthyin a new gene on chromosome 5q33 in a new form of autosomal recessive congenital ichthyosis. Hum Mol Genet 13: 2473–2482. 15317751 10.1093/hmg/ddh263

[5] Grall A, Guaguère E, Planchais S, Grond S, Bourrat E, et al (2012) PNPLA1 mutations cause autosomal recessive congenital ichthyosis in golden retriever dogs and humans. Nat Genet 44: 140–147 doi:10.1038/ng.1056 22246504 10.1038/ng.1056

[6] Huber M, Rettler I, Bernasconi K, Frenk E, Lavrijsen SP, et al (1995) Mutations of keratinocyte transglutaminase in lamellar ichthyosis. Science 267: 525–528. 7824952 10.1126/science.7824952

[7] Fischer J (2009) Autosomal recessive congenital ichthyosis. J Invest Dermatol 129: 1319–1321 doi:10.1038/jid.2009.57 19434086 10.1038/jid.2009.57

[8] Morales J, Al-Sharif L, Khalil DS, Shinwari JM a, Bavi P, et al (2009) Homozygous mutations in ADAMTS10 and ADAMTS17 cause lenticular myopia, ectopia lentis, glaucoma, spherophakia, and short stature. Am J Hum Genet 85: 558–568 doi:10.1016/j.ajhg.2009.09.011 19836009 10.1016/j.ajhg.2009.09.011 PMC2775842

[9] Farias FHG, Johnson GS, Taylor JF, Giuliano E, Katz ML, et al (2010) An ADAMTS17 splice donor site mutation in dogs with primary lens luxation. Invest Ophthalmol Vis Sci 51: 4716–4721. 20375329 10.1167/iovs.09-5142

[10] Schwarz JM, Rödelsperger C, Schuelke M, Seelow D (2010) MutationTaster evaluates disease-causing potential of sequence alterations. Nat Methods 7: 575–576 doi:10.1038/nmeth0810-575 20676075 10.1038/nmeth0810-575

[11] Mizutani Y, Kihara A, Igarashi Y (2006) LASS3 (longevity assurance homologue 3) is a mainly testis-specific (dihydro)ceramide synthase with relatively broad substrate specificity. Biochem J 398: 531–538. 16753040 10.1042/BJ20060379 PMC1559458

[12] Jennemann R, Rabionet M, Gorgas K, Epstein S, Dalpke A, et al (2012) Loss of ceramide synthase 3 causes lethal skin barrier disruption. Hum Mol Genet 21: 586–608 doi:10.1093/hmg/ddr494 22038835 10.1093/hmg/ddr494

[13] Vielhaber G, Pfeiffer S, Brade L, Lindner B, Goldmann T, et al (2001) Localization of ceramide and glucosylceramide in human epidermis by immunogold electron microscopy. J Invest Dermatol 117: 1126–1136 doi:10.1046/j.0022-202x.2001.01527.x 11710923 10.1046/j.0022-202x.2001.01527.x

[14] Nagy E, Maquat LE (1998) A rule for termination-codon position within intron-containing genes: when nonsense affects RNA abundance. Trends Biochem Sci 23: 198–199. 9644970 10.1016/s0968-0004(98)01208-0

[15] Gillingham AK, Munro S (2003) Long coiled-coil proteins and membrane traffic. Biochimica et Biophysica Acta (BBA) - Molecular Cell Research 1641: 71–85 doi:10.1016/S0167-4889(03)00088-0 12914949 10.1016/s0167-4889(03)00088-0

[16] Mason JM, Arndt KM (2004) Coiled coil domains: stability, specificity, and biological implications. Chembiochem 5: 170–176 doi:10.1002/cbic.200300781 14760737 10.1002/cbic.200300781

[17] Harbury PH, Kim PS, Alber T (1994) Crystal structure of an isoleucine-zipper trimer. Nature 371: 80–83. 8072533 10.1038/371080a0

[18] Mahrenholz CC, Abfalter IG, Bodenhofer U, Volkmer R, Hochreiter S (2011) Complex networks govern coiled-coil oligomerization–predicting and profiling by means of a machine learning approach. Mol Cell Proteomics 10: M110.004994 doi:10.1074/mcp.M110.004994 21311038 10.1074/mcp.M110.004994 PMC3098589

[19] Venkataraman K, Riebeling C, Bodennec J, Riezman H, Allegood JC, et al (2002) Upstream of growth and differentiation factor 1 (uog1), a mammalian homolog of the yeast longevity assurance gene 1 (LAG1), regulates N-stearoyl-sphinganine (C18-(dihydro)ceramide) synthesis in a fumonisin B1-independent manner in mammalian cells. J Biol Chem 277: 35642–35649. 12105227 10.1074/jbc.M205211200

[20] Guillas I, Jiang JC, Vionnet C, Roubaty C, Uldry D, et al (2003) Human homologues of LAG1 reconstitute Acyl-CoA-dependent ceramide synthesis in yeast. J Biol Chem 278: 37083–37091. 12869556 10.1074/jbc.M307554200

[21] Riebeling C, Allegood JC, Wang E, Merrill AH, Futerman AH (2003) Two mammalian longevity assurance gene (LAG1) family members, trh1 and trh4, regulate dihydroceramide synthesis using different fatty acyl-CoA donors. J Biol Chem 278: 43452–43459. 12912983 10.1074/jbc.M307104200

[22] Levy M, Futerman AH (2010) Mammalian ceramide synthases. IUBMB Life 62: 347–356 doi:10.1002/iub.319 20222015 10.1002/iub.319 PMC2858252

[23] Stiban J, Tidhar R, Futerman AH (2010) Ceramide synthases: roles in cell physiology and signaling. Adv Exp Med Biol 688: 60–71. 20919646 10.1007/978-1-4419-6741-1_4

[24] Mizutani Y, Kihara A, Igarashi Y (2005) Mammalian Lass6 and its related family members regulate synthesis of specific ceramides. Biochem J 390: 263–271. 15823095 10.1042/BJ20050291 PMC1184580

[25] Laviad EL, Albee L, Pankova-Kholmyansky I, Epstein S, Park H, et al (2008) Characterization of ceramide synthase 2: tissue distribution, substrate specificity, and inhibition by sphingosine 1-phosphate. J Biol Chem 283: 5677–5684. 18165233 10.1074/jbc.M707386200

[26] Lahiri S, Futerman AH (2005) LASS5 is a bona fide dihydroceramide synthase that selectively utilizes palmitoyl-CoA as acyl donor. J Biol Chem 280: 33735–33738. 16100120 10.1074/jbc.M506485200

[27] Rabionet M, Van der Spoel AC, Chuang C-C, Von Tümpling-Radosta B, Litjens M, et al (2008) Male germ cells require polyenoic sphingolipids with complex glycosylation for completion of meiosis: a link to ceramide synthase-3. J Biol Chem 283: 13357–13369. 18308723 10.1074/jbc.M800870200 PMC2442322

[28] Mizutani Y, Kihara A, Chiba H, Tojo H, Igarashi Y (2008) 2-Hydroxy-ceramide synthesis by ceramide synthase family: enzymatic basis for the preference of FA chain length. J Lipid Res 49: 2356–2364 doi:10.1194/jlr.M800158-JLR200 18541923 10.1194/jlr.M800158-JLR200

[29] Mizutani Y, Mitsutake S, Tsuji K, Kihara A, Igarashi Y (2009) Ceramide biosynthesis in keratinocyte and its role in skin function. Biochimie 91: 784–790 doi:10.1016/j.biochi.2009.04.001 19364519 10.1016/j.biochi.2009.04.001

[30] Feingold KR (2007) Thematic review series: skin lipids. The role of epidermal lipids in cutaneous permeability barrier homeostasis. J Lipid Res 48: 2531–2546. 17872588 10.1194/jlr.R700013-JLR200

[31] Uchida Y, Holleran WM (2008) Omega-O-acylceramide, a lipid essential for mammalian survival. J Dermatol Sci 51: 77–87 doi:10.1016/j.jdermsci.2008.01.002 18329855 10.1016/j.jdermsci.2008.01.002

[32] Aldahmesh MA, Mohamed JY, Alkuraya HS, Verma IC, Puri RD, et al (2011) Recessive mutations in ELOVL4 cause ichthyosis, intellectual disability, and spastic quadriplegia. Am J Hum Genet 89: 745–750. 22100072 10.1016/j.ajhg.2011.10.011 PMC3234380

[33] Vasireddy V, Uchida Y, Salem N, Kim SY, Mandal MNA, et al (2007) Loss of functional ELOVL4 depletes very long-chain fatty acids (> or = C28) and the unique omega-O-acylceramides in skin leading to neonatal death. Hum Mol Genet 16: 471–482. 17208947 10.1093/hmg/ddl480 PMC1839956

[34] McMahon A, Butovich I a, Mata NL, Klein M, Ritter R, et al (2007) Retinal pathology and skin barrier defect in mice carrying a Stargardt disease-3 mutation in elongase of very long chain fatty acids-4. Mol Vis 13: 258–272. 17356513 PMC2633486

[35] Uchida Y, Cho Y, Moradian S, Kim J, Nakajima K, et al (2010) Neutral lipid storage leads to acylceramide deficiency, likely contributing to the pathogenesis of Dorfman-Chanarin syndrome. J Invest Dermatol 130: 2497–2499 doi:10.1038/jid.2010.145 20520629 10.1038/jid.2010.145

[36] Radner FPW, Streith IE, Schoiswohl G, Schweiger M, Kumari M, et al (2010) Growth retardation, impaired triacylglycerol catabolism, hepatic steatosis, and lethal skin barrier defect in mice lacking comparative gene identification-58 (CGI-58). J Biol Chem 285: 7300–7311 doi:10.1074/jbc.M109.081877 20023287 10.1074/jbc.M109.081877 PMC2844178

[37] Jennemann R, Kaden S, Sandhoff R, Nordström V, Wang S, et al (2012) Glycosphingolipids are essential for intestinal endocytic function. J Biol Chem 287: 32598–32616. 22851168 10.1074/jbc.M112.371005 PMC3463339

[38] Furuse M, Hata M, Furuse K, Yoshida Y, Haratake A, et al (2002) Claudin-based tight junctions are crucial for the mammalian epidermal barrier: a lesson from claudin-1-deficient mice. J Cell Biol 156: 1099–1111 doi:10.1083/jcb.200110122 11889141 10.1083/jcb.200110122 PMC2173463

[39] Schempp W, Meer B (1983) Cytologic evidence for three human X-chromosomal segments escaping inactivation. Hum Genet 63: 171–174. 6682404 10.1007/BF00291539

[40] Schempp W, Binkele A, Arnemann J, Gläser B, Ma K, et al (1995) Comparative mapping of YRRM- and TSPY-related cosmids in man and hominoid apes. Chromosome Res 3: 227–234. 7606360 10.1007/BF00713047

[41] Ried T, Baldini A, Rand TC, Ward DC (1992) Simultaneous visualization of seven different DNA probes by in situ hybridization using combinatorial fluorescence and digital imaging microscopy. Proc Natl Acad Sci U S A 89: 1388–1392. 1741394 10.1073/pnas.89.4.1388 PMC48456

[42] Breiden B, Gallala H, Doering T, Sandhoff K (2007) Optimization of submerged keratinocyte cultures for the synthesis of barrier ceramides. Eur J Cell Biol 86: 657–673 doi:10.1016/j.ejcb.2007.02.006 17714827 10.1016/j.ejcb.2007.02.006

[43] Wertz PW, Miethke MC, Long SA, Strauss JS, Downing DT (1985) The composition of the ceramides from human stratum corneum and from comedones. J Invest Dermatol 84: 410–412. 3158712 10.1111/1523-1747.ep12265510

