# Correction: Mutations in *CERS3* Cause Autosomal Recessive Congenital Ichthyosis in Humans

**DOI:** 10.1371/annotation/df5af830-8e1d-495a-a206-f881ed85e7fe

**Published:** 2013-06-28

**Authors:** Franz P. W. Radner, Slaheddine Marrakchi, Peter Kirchmeier, Gwang-Jin Kim, Florence Ribierre, Bourane Kamoun, Leila Abid, Michael Leipoldt, Hamida Turki, Werner Schempp, Roland Heilig, Mark Lathrop, Judith Fischer

The following information was missing from the Funding section: "This work has been supported in part by grants to M. Lathrop from Genome Québec, le Ministère de l'Enseignement supérieur, de la Recherche, de la Science et de la Technologie (MESRST) Québec, and from the ANR in France for the Labex project "Medical Genomics".

