# Supplementary figures and images for: Mutations in CERS3 Cause Autosomal Recessive Congenital Ichthyosis in Humans
Source: PLoS Genet. 2013 Jun 6;9(6):e1003536. doi: 10.1371/journal.pgen.1003536 (PMC3675029; doi:10.1371/journal.pgen.1003536)

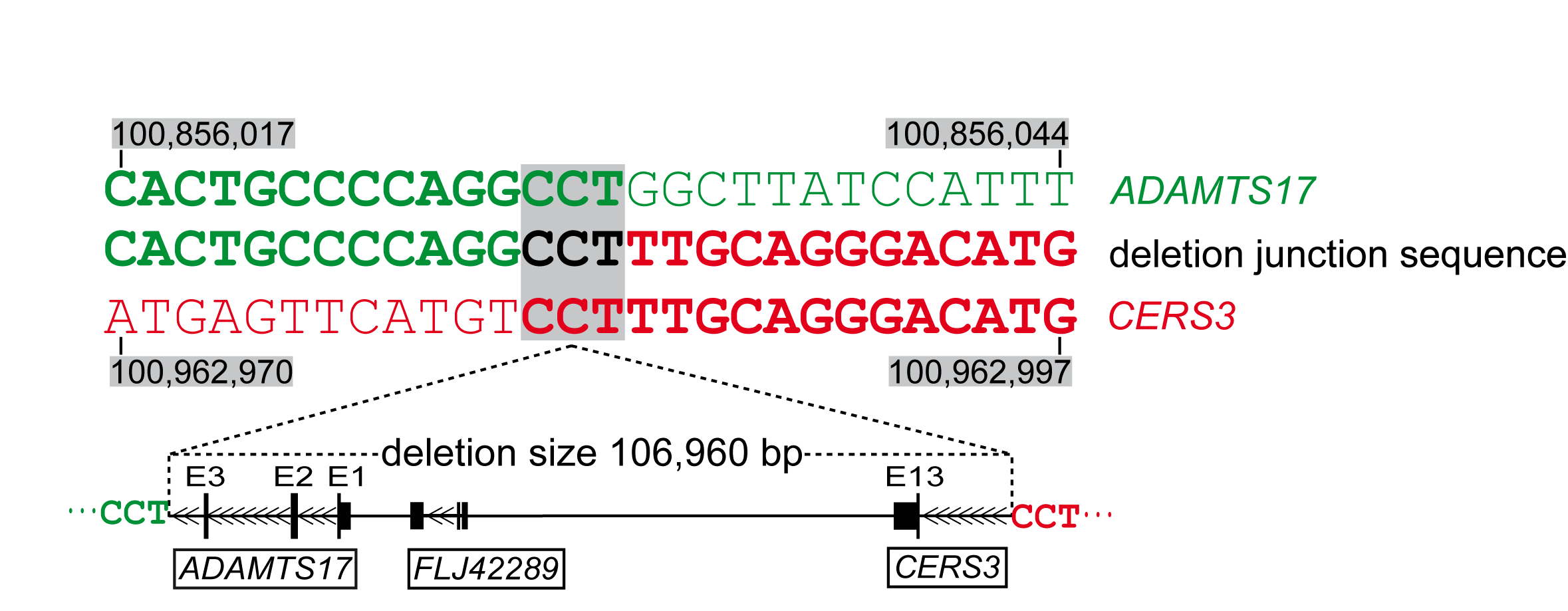

Supplement: Figure S1 — Detailed breakpoint information and deleted elements in patients D1, D2, C, and S. The upper panel shows the wild-type ADAMTS17 sequence in green, aligned with the deletion junction sequence in green and red, and the wild-type CERS3 sequence in red. The coordinates of both sequences on chromosome 15 are shown according to UCSC hg19, February 2009. The junction sequence is indicated by bold characters corresponding to ADAMTS17 (left part in green) and CERS3 (right part in red). The junctional CCT is highlighted in grey. The lower panel illustrates the deleted region containing 5′UTR and exon 1–3 of ADAMTS17, the complete sequence of FLJ42289, and exon 13 of CERS3 with 3′UTR. (TIF) [file pgen.1003536.s001.tif]

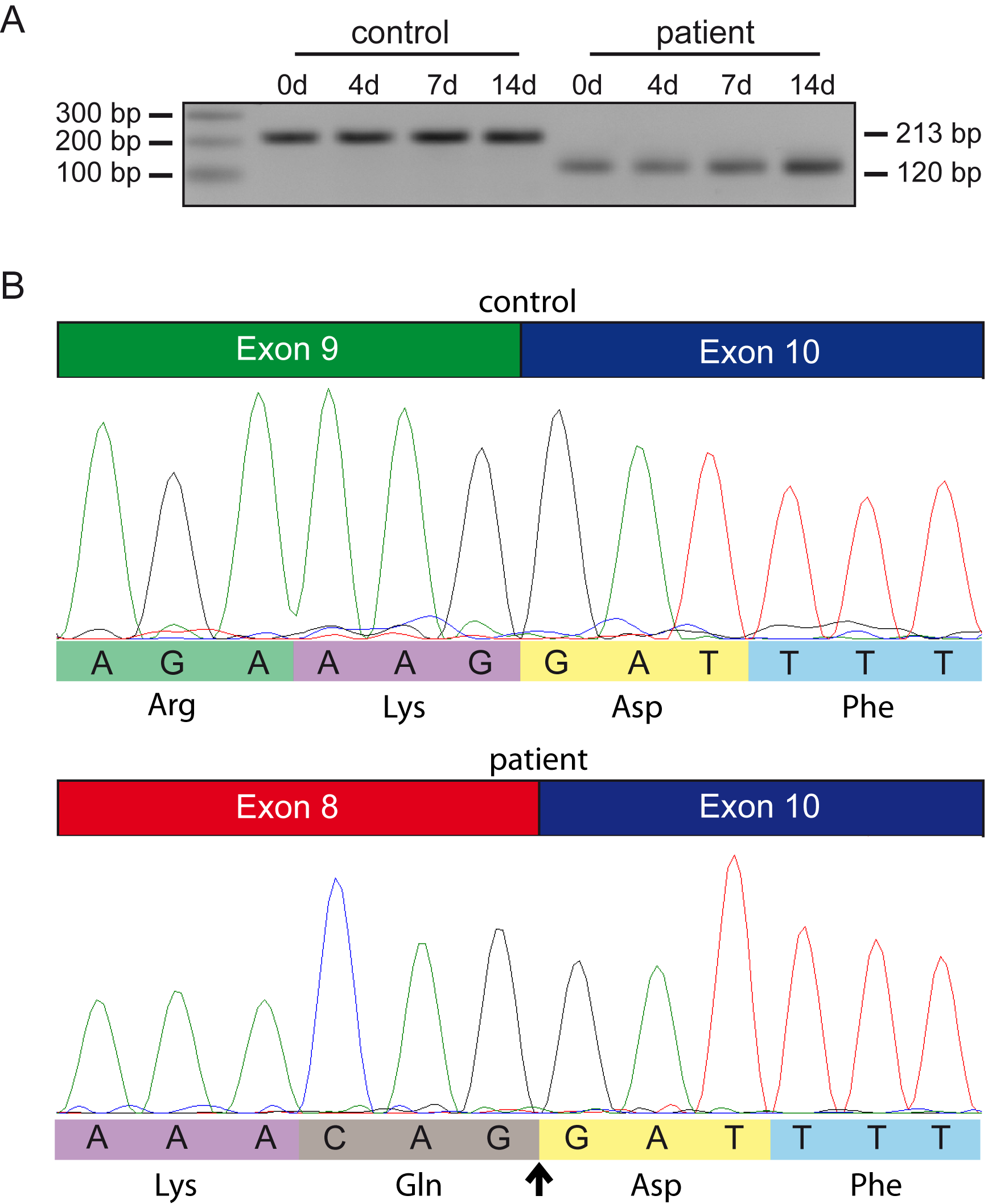

Supplement: Figure S2 — RT-PCR analysis and sequencing of CERS3 cDNA. (A) RT-PCR analysis of CERS3 mRNA from control and patient H keratinocytes before differentiation (0 d) and at day 4, 7, and 14 after induction of differentiation. Primers located in exon 8 and 10 of CERS3 were used to determine the splicing pattern. The 213 bp-sized DNA fragment from control keratinocytes corresponds to the full-length CERS3 coding transcript. The 120 bp-sized DNA fragment of patient H keratinocytes represent a novel CERS3 coding transcript lacking exon 9 due to the splice donor site mutation of exon 9 (c.609+1G>T). (B) The sequencing of CERS3 cDNA from healthy control and patient keratinocytes, which were differentiated in vitro for 14 days revealed an in-frame deletion of exon 9 in patient H (indicated by an arrow). (TIF) [file pgen.1003536.s002.tif]

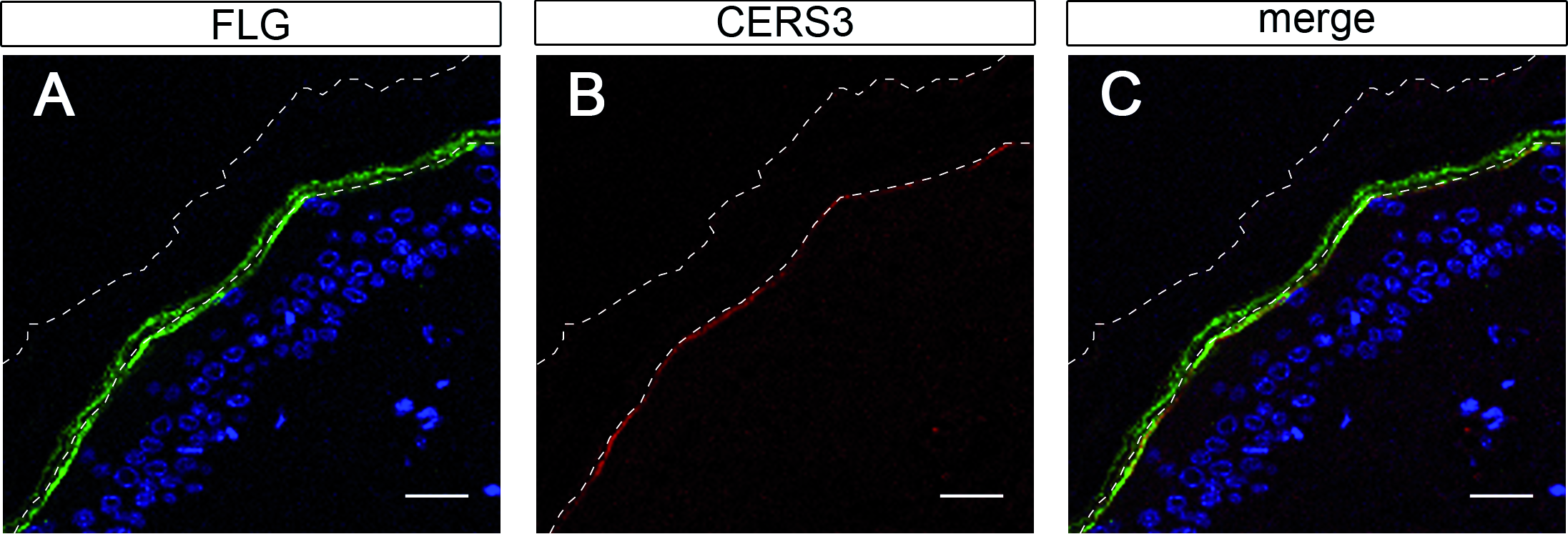

Supplement: Figure S3 — Localization of CERS3 protein in human healthy skin. Confocal microscopy images of double immunostained human skin paraffin section of a healthy individual for (A) the stratum granulosum marker FLG (filaggrin) (green) with DAPI as nuclear counterstaining (blue) and for (B) CERS3 (red), which localizes to a narrowly restricted apical layer of the epidermis. (C) The merged picture shows the co-localization of CERS3 and filaggrin at the interface between the stratum granulosum and the stratum corneum in the epidermis. The thin dashed lines indicate the interface between the stratum granulosum and the stratum corneum as well as the upper edge of the stratum corneum. Scale bars, 25 µm. (TIF) [file pgen.1003536.s003.tif]

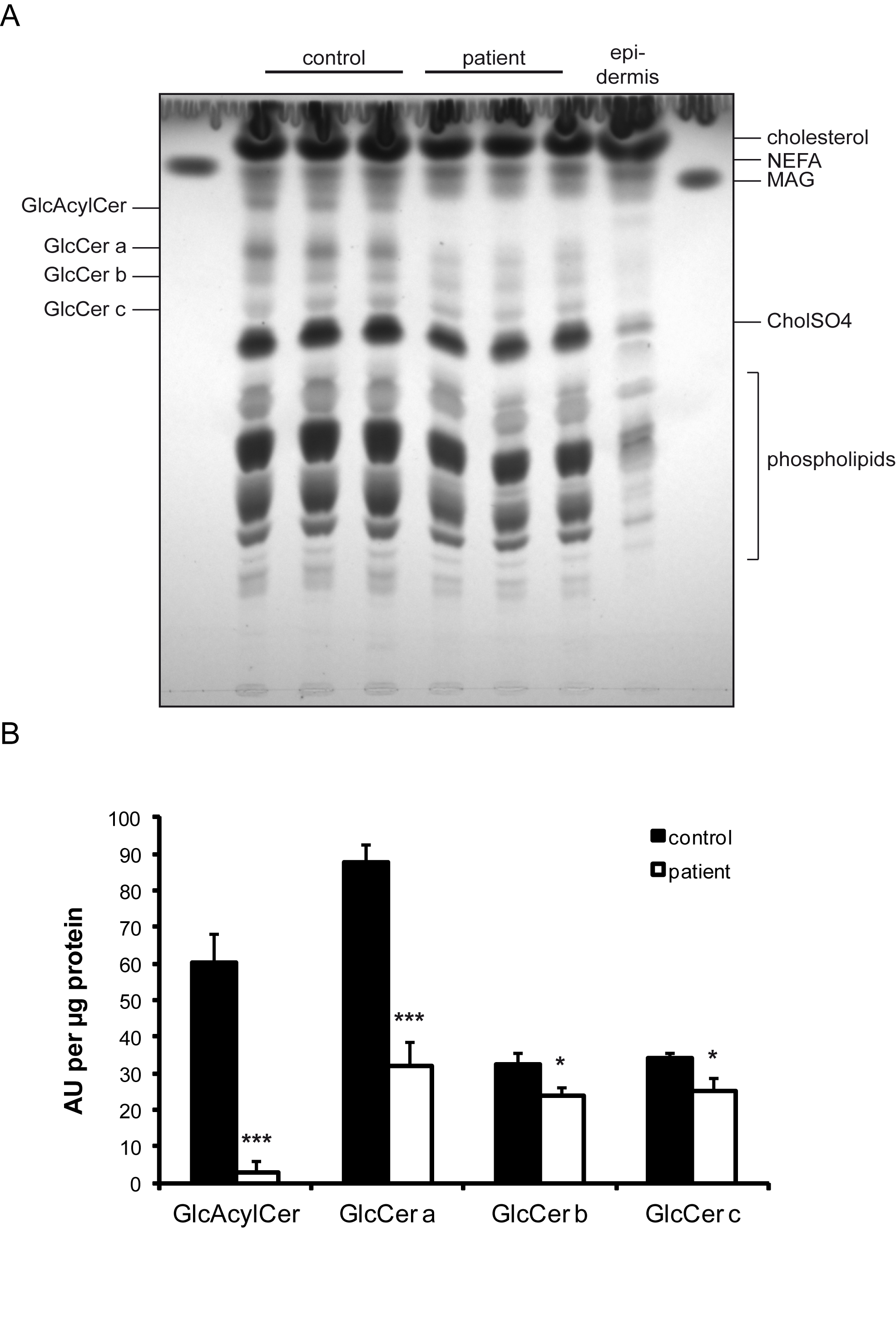

Supplement: Figure S4 — TLC analysis of lipid extracts from healthy control and patient H keratinocytes 14 days after induction of differentiation. (A) Lipids corresponding to 400 µg of cellular protein were extracted from cultures, separated by TLC using chloroform/methanol/water (70/30/5 v/v/v), and quantified after carbonization. Polar lipids (GlcCer and GlcAcylCer) were identified according to Breiden et al. [42] using an epidermal lipid extract of a healthy control individual as reference. (B) Data are presented as mean values +S.D. of triplicate samples and are representative for three independent experiments. Statistical significance was determined by unpaired two-tailed Student's t-test (* p<0.05, *** p<0.001). Abbreviations: CholSO4, cholesterol sulfate; GlcAcylCer, glucosylacylceramides; GlcCer, glucosylceramides; MAG, monoacylglycerols; NEFA, non-esterified fatty acids. (TIF) [file pgen.1003536.s004.tif]
