# Supplementary material for: Mutations in CERS3 Cause Autosomal Recessive Congenital Ichthyosis in Humans
Source: PLoS Genet. 2013 Jun 6;9(6):e1003536. doi: 10.1371/journal.pgen.1003536 (PMC3675029; doi:10.1371/journal.pgen.1003536)
Supplement: Text S1 — Detailed clinical descriptions of Tunisian patients D1, D2, S, C, and H are included in Text S1. (DOC) [file pgen.1003536.s005.doc]

**Supplementary Information**

**Clinical description of Tunisian patients (D1, D2, S, C, and H)**

Four patients from three consanguineous Tunisian families were followed for similar presentation of a syndromic ichthyosis characterized by a congenital ichthyosiform erythroderma associated with abnormalities of eyes, heart, and bones. One additional patient (H) with non-syndromic ARCI was also examined (Figure 1 and Table 1).

**Patient D1:** This 37-year-old man was born with a collodion membrane, which evolved toward ichthyosiform erythroderma accentuated on his face, but without ectropion of the eyelids. He is of short stature (1.65 m) and thickset, without mental impairment. He has generalized ichthyosis with fine, white scaling on the face, trunk, and arms and larger brownish scales on the legs. On the back of his hands the skin was thickened and had a peculiar progeroid appearance. The palms and soles showed hyperlinearity with moderate hyperkeratosis in zones of plantar pressure. He presented stiffness of fingers and brachydactyly.

Previously, this patient had undergone surgery without implantation for microspherophakia, which affected both eyes 22 years ago. One year afterwards, cataract surgery was complicated by detachment of the retina of the left eye. This detachment was treated by three interventions on this eye with therapeutic failure and loss of function of this eye. The patient has been treated for secondary glaucoma for 10 years in his right eye, but compliance was insufficient. On clinical examination, his left eye showed a divergence of the eye globe. In the right eye, there was severe glaucoma with negative perception of light, transparent cornea, scleral ectasia, and areflectic semi-mydriasis with balanced tonus under treatment. The fundus had a flat retina with optic atrophy as a sequela to his secondary glaucoma.

At the age of 35 years, the patient began to present episodes of tachycardia. Holter recording revealed atrial tachycardia. The transthoracic echocardiography showed dilated left atrium (surface of the LA 21 cm²), a dysplastic mitral valve, and dilated cardiomyopathy with altered systolic function (LVEF; left ventricular ejection fraction = 45%). Electrocardiographic characteristics were as following: PR duration = 0.16 sec, QRS = 0.08, QTc = 0.45 sec. There was no disturbance of polarization.

**Patient D2**: This patient is a small stocky man (1.63 m) born as a collodion baby, aged 22 years at present. He presented ichthyosiform erythroderma, with accentuated erythema of the face. The scales are brownish and large, discrete at the borders of the scalp and more accentuated on the limbs and trunk. The palmoplantar skin exhibits hyperlinearity with discrete hyperkeratosis of the zones of plantar pressure. There is brachydactyly of both hands. Visual acuity on ophthalmologic examination was 3/10 in the right eye, corrected to 9/10 with a correction for myopia of -9. Visual acuity in the left eye without correction was 4/10 and 10/10 after correction for myopia of -3.75. Ocular tonus was at the upper limit for the right eye (18 mmHg) and normal in the left eye (15 mmHg). There was bilateral iridonesis with a shallow anterior chamber, especially in the right eye. There was bilateral microspherophakia with right ectopia. The fundus was normal, the field of vision was normal and B echography was normal with an axial length of 21.8 on the right and 22 on the left. There were no cardiac symptoms. Systemic transthoracic echocardiography showed a mitral valve dysplasia. The other valves were normal as well as the ejection fraction (LVEF), which was 60%.

Patient D1 and D2 belong to the same family (D1 is the uncle of D2).

**Patient S:** This 14-year-old boy was born as a collodion baby. He is small (1.50 m) and stocky. He presents ichthyosiform erythroderma with erythema most intense on the face. The scales are fine and whitish on his face, trunk, and arms, but large and white on his legs. His palms present hyperlinearity, whereas the cutaneous pattern of the back of the hands is accentuated, resulting in a progeroid appearance. The soles show hyperkeratosis. Multiple nevi are disseminated over the face, trunk, and limbs. Brachydactyly and stiffness of the fingers were present. The patient has been undergoing ophthalmological treatment since the age of 4 years. He has an optical correction and has been treated for amblyopia of the right eye. On examination, there was a pronounced myopia of -6 in both eyes with visual acuity of 8/10 in the right eye and 10/10 in the left eye, associated with isolated bilateral microspherophakia without ectopia or hypertonia. A shallow anterior chamber was seen on both sides. Systematic trans-thoracic echocardiography did not reveal any valve abnormalities. The LVEF was 60%.

**Patient C:** This 11-year-old boy was born as a collodion baby. Despite his rather short stature (1.33 m), he was not stocky. At the moment he presents a discrete erythema of the skin with ichthyosis consisting of small white scales on his face, trunk, and arms. The scales are larger on his legs. Palmoplantar hyperlinearity and moderate hyperkeratosis of the soles were likewise present. The back of the hands show premature skin aging. Multiple melanocytic nevi with predominance on his hands were also noted. There was no brachydactyly or stiffness of fingers. On ophthalmologic examination the visual acuity was 10/10 in the right eye with a correction for astigmatism of 2 diopters and myopia of -8 in the right eye and 10/10 in the left eye with -7.25. Examination shows bilateral microspherophakia without ectopia and normal ocular tonus and fundus. Transthoracic echocardiography revealed a dysplasic mitral valve. The other valves were normal as well as the LVEF, which was 60%.

**Patient H:** This 30-year-old woman progressed to an ichthyosiform erythroderma after being born as a collodion baby. The erythema is pronounced on her face. Fine whitish scales are present over the whole body with the exception of the legs where they are large and brownish. Moderate hyperlinearity is found on her palms, whereas yellowish plantar keratoderma is present on the pressure zones. Multiple melanocytic nevi are found on the back of her hands. There is no brachydactyly or stiffness of the fingers. The results of ophthalmologic examination were normal. The patient had no cardiac symptoms and the echocardiographic examination did not show any abnormalities.
